# A case of Arnold-Chiari malformation type 2

**DOI:** 10.11604/pamj.2023.44.115.36848

**Published:** 2023-03-01

**Authors:** Utkarsha Khaire, Dnyanesh Joshi

**Affiliations:** 1 Department of Samhita and Siddhant, Mahatma Gandhi Ayurved College, Hospital & Research Centre, Datta Meghe Institute of Medical Sciences (Deemed To Be University) Salod (H), Wardha, Maharashtra, India

**Keywords:** The Chiari type II malformation, myelomeningocele, spinal astrocytoma

## Image in medicine

The Chiari type II malformation (Arnold-Chiari malformation) is a complex congenital malformation of the brain, nearly always associated with myelomeningocele, and the most common serious malformation of the posterior fossa. This condition has skull, dural, brain, spinal, and spinal cord manifestations, including downward displacement of the medulla, fourth ventricle, and cerebellum into the cervical spinal canal, as well as elongation of the pons and fourth ventricle, probably due to a relatively small posterior fossa.

**Figure 1:**
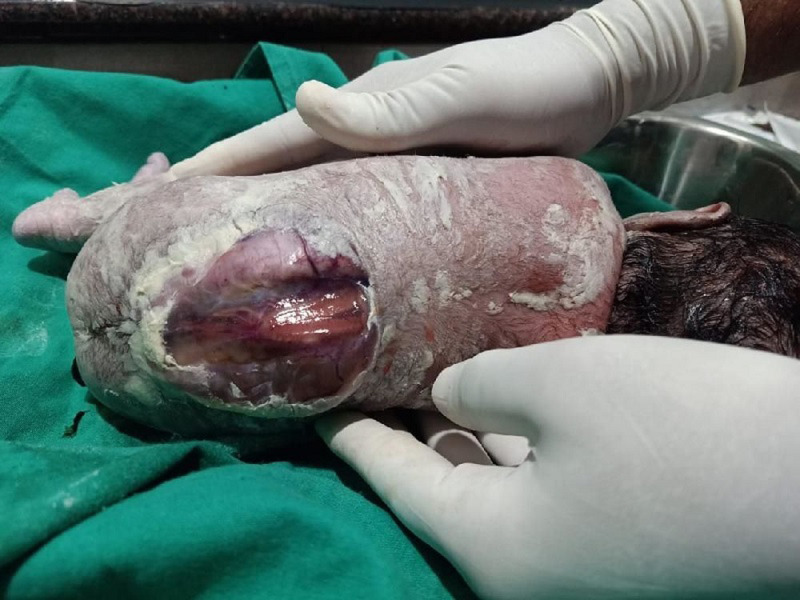
Arnold-Chiari malformation type II

